# Total intracranial hemorrhage volume measurement summating all compartments best in traumatic and nontraumatic intracranial bleeding

**DOI:** 10.1002/brb3.3481

**Published:** 2024-04-28

**Authors:** MacKenzie Horn, Ankur Banerjee, Linda Kasickova, Ondrej Volny, Hyun Seok Choi, Federica Letteri, Tomoyuki Ohara, Koji Tanaka, Stuart Connolly, Per Ladenvall, Mark Crowther, Jan Beyer‐Westendorf, Ashkan Shoamanesh, Andrew M. Demchuk, Abdulaziz S. Al Sultan, for the ANNEXa‐4 Investigators

**Affiliations:** ^1^ Department of Clinical Neurosciences University of Calgary Calgary Canada; ^2^ Department of Radiology University of Calgary Calgary Canada; ^3^ Department of Medicine, Division of Neurology University of Alberta Edmonton Canada; ^4^ Faculty of Medicine Masaryk University Brno Czech Republic; ^5^ Department of Neurology University Hospital Ostrava Ostrava Czech Republic; ^6^ Czech National Centre for Evidence‐Based Healthcare and Knowledge Translation, Faculty of Medicine Masaryk University Brno Czech Republic; ^7^ International Clinical Research Center (ICRC) St. Anne's University Hospital Brno Czech Republic; ^8^ Department of Radiology Seoul Medical Center Seoul South Korea; ^9^ Unit of Neurology, San Jacopo Hospital Pistoia Italy; ^10^ Department of Neurology Kyoto Prefectural University of Medicine Kyoto Japan; ^11^ Department of Medicine McMaster University Hamilton Ontario Canada; ^12^ Biopharmaceuticals R&D, AstraZeneca Mölndal Sweden; ^13^ University Hospital Carl Gustav Carus Dresden Dresden Germany; ^14^ Department of Medicine, Division of Neurology Royal Columbian Hospital New Westminster Canada

**Keywords:** neuroimaging, neuroscience, neurology, stroke

## Abstract

**Background and purpose:**

The ANNEXA‐4 trial measured hemostatic efficacy of andexanet alfa in patients with major bleeding taking factor Xa inhibitors. A proportion of this was traumatic and nontraumatic intracranial bleeding. Different measurements were applied in the trial including volumetrics to assess for intracranial bleeding depending on the compartment involved. We aimed to determine the most reliable way to measure intracranial hemorrhage (ICrH) volume by comparing individual brain compartment and total ICrH volume.

**Methods:**

Thirty patients were randomly selected from the ANNEXA‐4 database to assess measurement of ICrH volume by compartment and in total. Total and compartmental hemorrhage volumes were measured by five readers using Quantomo software. Each reader measured baseline hemorrhage volumes twice separated by 1 week. Twenty‐eight different ANNEXA‐4 subjects were also randomly selected to assess intra‐rater reliability of total ICrH volume measurement change at baseline and 12‐h follow up, performed by three readers twice to assess hemostatic efficacy categories used in ANNEXA‐4.

**Results:**

Compartmental minimal detectable change percentages (MDC%) ranged between 9.72 and 224.13, with the greatest measurement error occurring in patients with a subdural hemorrhage. Total ICrH volume measurements had the lowest MDC%, which ranged between 6.57 and 33.52 depending on the reader.

**Conclusion:**

Measurement of total ICrH volumes is more accurate than volume by compartment with less measurement error. Determination of hemostatic efficacy was consistent across readers, and within the same reader, as well as when compared to consensus read. Volumetric analysis of intracranial hemostatic efficacy is feasible and reliable when using total ICrH volumes.

## Graphical abstract

Measurement of total ICrH volumes is more accurate than volume by compartment with less measurement error. Determination of hemostatic efficacy was consistent across readers, and within the same reader, as well as when compared to consensus read. Volumetric analysis of intracranial hemostatic efficacy is feasible and reliable when using total intracranial volumes.

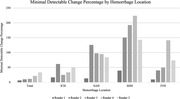

## 1 INTRODUCTION

Intracerebral hemorrhage is associated with a poor prognosis for patients, with high rates of mortality (Fogelholm et al., 2005; Sacco et al., 2009; Zahuranec et al., 2014) and poor health‐related quality of life (Christensen et al., 2009). Hematoma expansion—or an increase in intracerebral hemorrhage volume over time—is an established predictor of poor outcome in patients with intracerebral hemorrhage (Davis et al., 2006). As such, accurate measurement of intracerebral hemorrhage volumes can serve an important role in the prognostication of clinical outcomes in patients with intracerebral hemorrhage. The search for prediction tools of clinical outcomes for patients with intracerebral hemorrhage has led to the development of a number of scoring systems which rely on quantifying/characterizing hemorrhage volumes (Brouwers et al., 2014; Hemphill et al., 2001; Rost et al., 2008). Hematoma expansion in spontaneous and traumatic intracranial hemorrhage (ICrH) involving other ICrH compartments (subdural, subarachnoid, and epidural) has been less studied and there exists limited information on methods of measuring ICrH volume in these compartments and their accuracy and reliability.

The ANNEXA‐4 trial measured the usefulness of andexanet alfa (andexanet) as a reversal agent for patients with acute major bleeding while taking factor Xa inhibitors. The majority of patients in ANNEXA‐4 had either intracranial or gastrointestinal bleeding (Connolly et al., 2016). In the trial, a comparison of ICrH volumes at baseline and at follow‐up after andexanet administration was used to determine hemostatic efficacy using Quantomo software (Cybertrials, Inc). Quantomo is a semiautomatic hemorrhage segmentation software that has been shown to have improved intra‐ and inter‐rater reliability in comparison to the manual ABC/2 method (Kosior et al., 2011) and has been used to measure intracerebral (intraparenchymal) hemorrhage volumes in other studies (Demchuk et al., 2012).

Anticoagulant reversal trials, including patients ICrH, such as ANNEXA‐4, have demonstrated the need for more data on how to best measure hemorrhage volume for other ICrH compartments (i.e., subdural and subarachnoid) or when there is multi‐compartmental intracranial bleeding. In multi‐compartmental ICrH, the total volume of intracranial bleeding can be measured, or individual compartments can be measured separately. In measurement of total volume, the entire volume of intracranial blood is measured irrespective of the hemorrhage compartment. In the compartmental approach, separate measurements are done for intraparenchymal hemorrhage (IPH), intraventricular hemorrhage (IVH), subarachnoid hemorrhage (SAH), and subdural hemorrhage (SDH).

There are certain challenges that arise when measuring hemorrhage volumes in patients with ICrH. For compartmental volume measurements, the analysis can be subjective as to which compartment is involved in hemorrhages that contiguously cross multiple compartments or due to distorted neuroanatomy from the compressive forces of the hematoma.

Despite these challenges, it has been shown that the minimal detectable difference (MDD) for intracerebral and IVH volume is below the expansion criteria of >6 mL growth in patients with hemorrhages <50 mL, with a larger MDD for larger hemorrhage volumes (Rodriguez‐Luna et al., 2016). These findings suggest that hemorrhage volumes can be calculated using Quantomo software within a reasonable margin of error.

The aim of our study is twofold: (1) to determine if hemostatic efficacy is most accurately assessed in patients with multi‐compartmental ICrH through measuring total volume (sum of all ICrH compartments) or by compartment and (2) to assess if total ICrH volume measurement performed at baseline and follow‐up can achieve a high degree of intra‐rater reliability for categorizing hemostatic efficacy.

## 2 METHODS

All subjects were patients with intracranial bleeds, including traumatic hemorrhages, who were enrolled in the ANNEXA‐4 trial.

To assess our first aim, 30 patients were randomly selected with multi‐compartmental bleeding from the ANNEXA‐4 database for analysis. Patients were selected from September 2015 to June 2017, had at least two of the following: IPH, IVH, SAH, or SDH, and had a CT scan at baseline presentation. A neuroradiologist, a stroke physician, a stroke fellow, a neurology resident, and a medical student measured hemorrhage volume at baseline and then remeasured the same set of scans 1 week later. Consensus read was determined by two senior neurologists who independently evaluated each image and then compared their measurements to reach a consensus. All hemorrhage volumes were measured using Quantomo, a semiautomatic hemorrhage volume measurement software. Minimal detectable change (MDC) and MDC% were calculated for each brain compartment and total ICrH volume for each reader with 95% confidence. The MDC% was determined using the following equation: (MDC/mean volume for the reader) × 100%.

To assess our second aim, 28 patients were randomly selected from the ANNEXA‐4 trial with either single or multi‐compartmental bleeds. These patients were independently selected from the trial database, and there was no overlap from the first set of 30 patients. Patients were enrolled in the trial between September 2015 and January 2019. Three readers (a stroke fellow, medical student, and research assistant) measured hemorrhage volume using Quantomo (Cybertrials, Inc) (Kosior et al., 2011). Readers measured hemorrhage volume on baseline imaging and 12‐h follow‐up imaging and then repeated this process 2 weeks later to minimize recall bias in the assessment of intra‐rater reliability. For each reader, Kappa Scores for both inter‐ and intra‐rater reliability for the categorization of hemostatic efficacy were calculated. Hemostatic efficacy was categorized into excellent, good, and poor efficacy if the hematoma expansion was a volumetric increase of ≤20%, >20% to ≤35%, and >35%, respectively, comparing follow up to baseline imaging.

## 3 RESULTS

### 3.1 Minimal detectable change by compartment and total ICrH study

**FIGURE 1:**
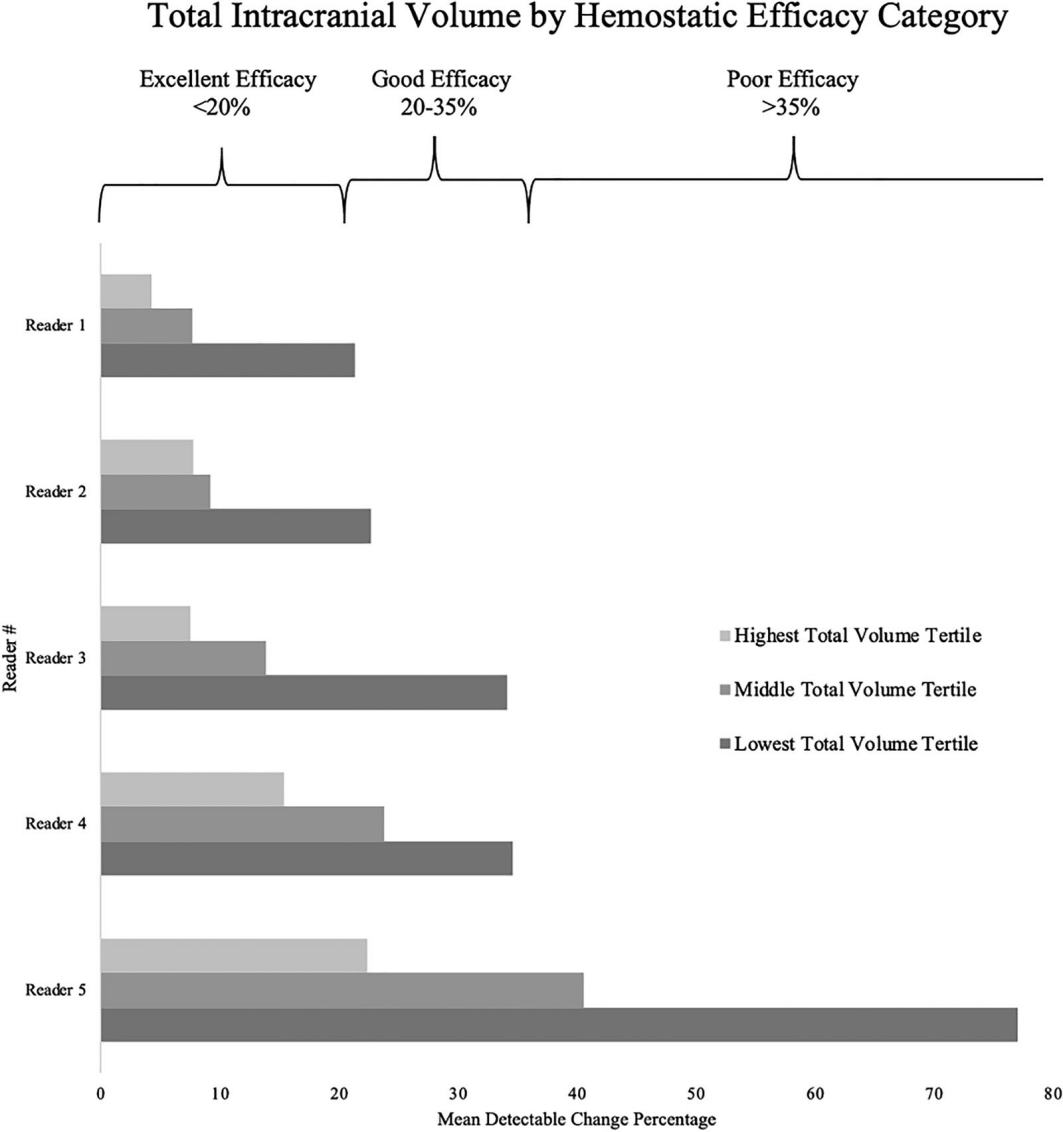
Total intracranial hemorrhage (ICrH) volume minimal detectable change (MDC) percentage by hemostatic efficacy.

**FIGURE 2:**
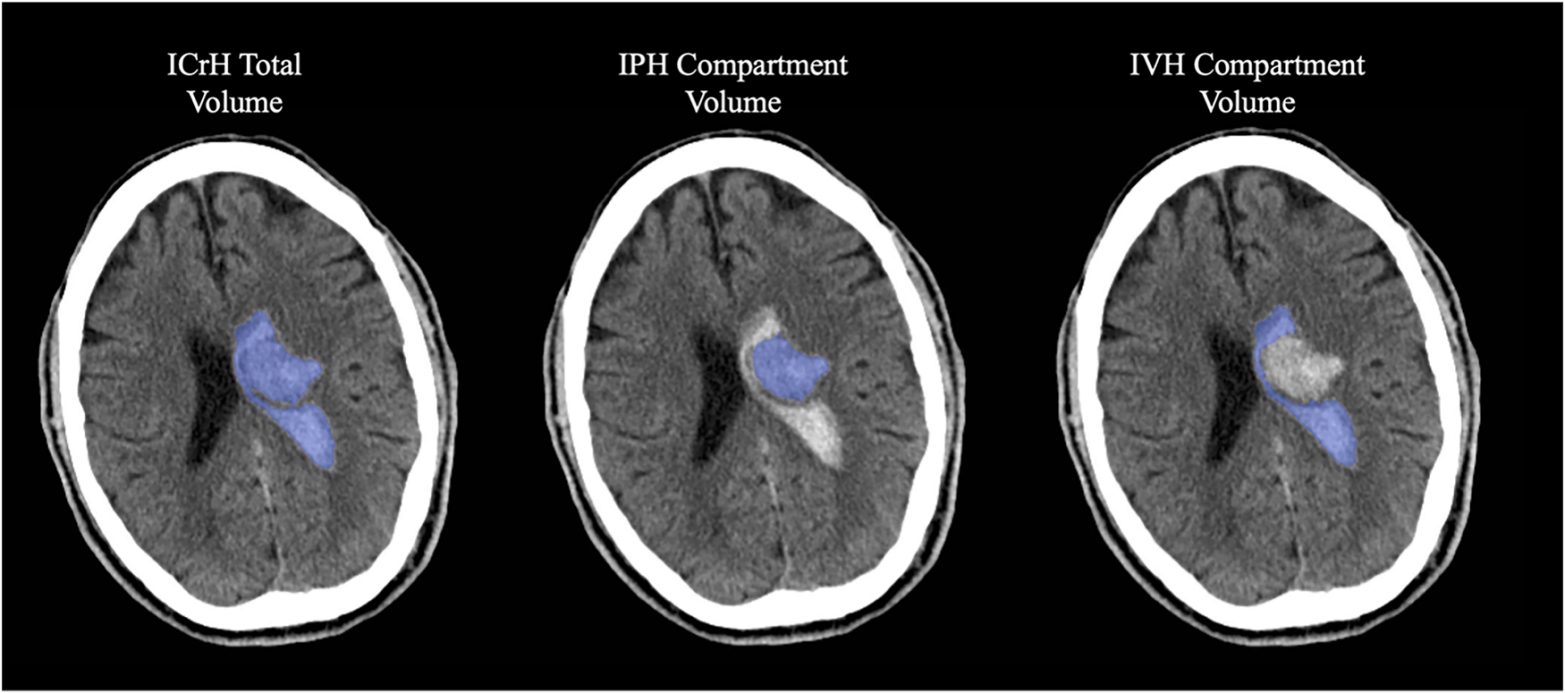
Quantomo measurement of intracranial hemorrhage (ICrH) versus hemorrhage volume by compartment.

MDC and MDC% for each reader were calculated by the individual brain compartment method and by the total ICrH volume method. Median total ICrH volume and compartmental volume with interquartile range as per consensus read and for each reader is summarized in Table 1. Although intraclass correlation coefficient for each compartment was higher than 91%, IPH MDC% were between 16.64 and 61.62, and IVH MDC% ranged between 9.72 and 141.53 among readers. SDH measurements were found to have the highest MDC%, which was between 39.43 and 224.13. SAH measurements also had high MDC%, which were calculated among the five readers ranging between 13.35 and 125.81. Total ICrH volume measurements had MDC% ranging between 6.57 and 33.52 (Figure 1). Figure 2 shows a case from this dataset using Quantomo software to measure total ICrH volume versus hemorrhage volume measurements by individual compartment.

**TABLE 1:** The volume of minimal detectable change (MDC_95_), their percentages stratified by volume in the different compartments and intracranial hemorrhage (ICrH) volume tertiles.

Intracranial hemorrhage (lower end–upper end)	Total ICrH SEM (mL) MDC (mL) MDC (%)	IPH SEM (mL) MDC (mL) MDC (%)	IVH SEM (mL) MDC (mL) MDC (%)	SDH SEM (mL) MDC (mL) MDC (%)	SAH SEM (mL) MDC (mL) MDC (%)
**Lowest tertile**	0.26–1.04	0.0–1.17	0.0–0.0	0.0–0.0	0.0–0.25
	0.72–2.89	0.0–3.25	0.0–0.0	0.0–0.0	0.0–0.70
	21.32–77.03	0.0–623.37	0.0–0.0	0.0–0.0	0.0–503.97
**Middle tertile**	0.63–3.06	0.40–3.82	0.04–0.20	0.02–0.76	0.22–1.00
	1.74–8.48	1.10–10.60	0.11–0.54	0.06–2.11	0.61–2.78
	7.68–40.55	16.64–153.90	146.74–357.18	177.09–262.24	72.28–374.81
**Highest tertile**	0.77–3.61	0.47–3.49	0.36–4.96	0.96–5.02	0.42–5.02
	2.13–10.02	1.30–9.66	0.98–13.75	2.65–13.92	1.17–13.92
	4.27–22.38	14.46–33.54	5.59–82.24	19.06–135.95	5.97–74.22
**All tertiles**	0.61–2.80	0.47–3.0	0.21–2.86	0.71–2.91	0.34–2.93
	1.69–7.76	1.30–8.32	0.57–7.94	1.96–8.07	0.95–8.12
	6.57–33.52	16.64–61.62	9.72–141.53	39.43–224.13	13.35–125.81

Abbreviations: IPH, intraparenchymal hemorrhage; IVH, intraventricular hemorrhage; SAH, subarachnoid hemorrhage; SDH, subdural hemorrhage; SEM, standard error of measurement.

### 3.2 Reliability of hemostatic efficacy determination using total intracranial hemorrhage volume

The hemostatic efficacy categories were determined by each of the three readers on two separate occasions and compared to consensus read. The consensus read categorized efficacy into poor in 29%, good in 7%, and excellent in 64%. Reader one categorized efficacy into poor in 36%–39%, good in 14%–29%, and excellent in 32%–50%. Reader two categorized efficacy into poor in 29%–32%, good in 7%–14%, and excellent in 54%–64%. Reader three categorized efficacy in exactly the same categories with both reads as the consensus read (Table 2). The overall kappa scores for hemostatic efficacy for inter‐rater reliability ranged from 0.35 to 1, and intra‐rater reliability ranged from 0.57 to 0.86 when using total ICrH volume.

**TABLE 2:** Hemostatic efficacy determination by three different readers.

Hemostatic efficacy category	Consensus (%)	Reader 1 1st read (%)	Reader 1 2nd read (%)	Reader 2 1st read (%)	Reader 2 2nd read (%)	Reader 3 1st read (%)	Reader 3 2nd read (%)
Poor	29	39	36	32	29	29	29
Good	7	29	14	14	7	7	7
Excellent	64	32	50	54	64	64	64

## 4 DISCUSSION

Multiple therapeutic trials are evaluating hemostatic agents to prevent or reduce ongoing brain bleeding. Accurate measurement of this ongoing brain bleeding is possible intracranially as a surrogate outcome of hemostasis by volumetric software analysis but is hampered (greater measurement error) when multiple intracranial compartments (i.e., subarachnoid, subdural, and intraparenchymal) are being measured separately. Measurement error when quantifying hematoma volumes could cloud the interpretation of hemostatic efficacy if the error of such measurements is too high. MDC is a method to evaluate this error. Definitions of excellent hemostatic efficacy of such treatments should be higher than the MDC of the measurement to be accurately detected. Although MDC has been used in multiple medical disciplines before (Dontje et al., 2018; Gavrielides et al., 2013; Katajapuu et al., 2020; Oda et al., 2010; Oxnard et al., 2011; Piedrahita‐Alonso et al., 2021), to our knowledge, this study is the first to establish ICrH volume MDC among readers.

**FIGURE 3:**
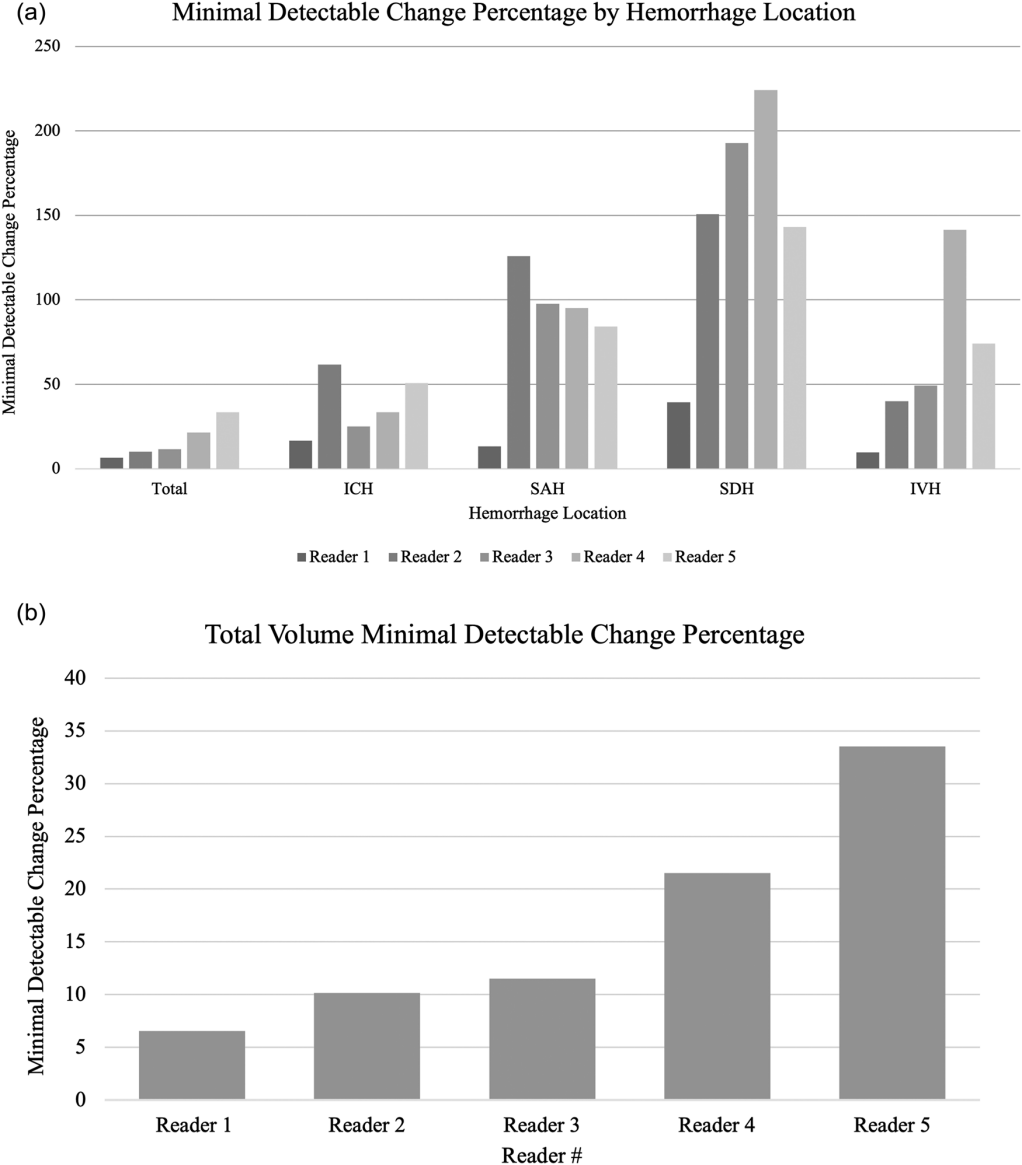
(a) Minimal detectable change (MDC) percentage by hemorrhage location. (b) MDC percentage for total intracranial hemorrhage volume.

Our study revealed that when measuring hemorrhage volume as a predictor of hemostatic efficacy, the measurement of total volume has a lower measurement error compared to measurements of individual compartments summed together, as demonstrated in Figure 3. Second, we demonstrate fair consistency between readers for categorizing these ICrH into specific hemostatic efficacy categories when using total ICrH volume, suggesting that this method may be advantageous for use in future studies, or potentially in clinical practice.

Volumetric segmentation by individual ICrH compartments was clearly shown in the study to be unreliable with very high measurement error (high MDC%). This is likely due to the difficulties separating the planes of each compartment when blood is present (i.e., IPH vs. IVH). SAHs had high measurement error likely in part due to the diffuse nature of such bleeding (hazy with unclear borders) and the difficulties distinguishing small areas of bleeding within the subarachnoid versus intraparenchymal compartment. Subdural hematomas had the worst measurement error which is likely confounded by multilobulated regions where uncoagulated blood (acute but hypodense), more chronic hemorrhage and hyperdense membrane (fibrous) tissue is present. We do not think distinction of planes and compartments can be easily solved by unique methodology. Fortunately, total ICrH volume was associated with relatively low measurement error as it removes the need for such distinction between planes and compartments. Despite the wide range in overall Kappa scores for inter‐ and intra‐rater reliability for the determination of hemostatic efficacy using total ICrH volume, when MDC% was measured by compartment versus total ICrH volume, there were substantially lower MDC% overall for total ICrH. This suggests that the measurement of total ICrH volume is a more precise estimate of hemostatic efficacy and therefore is the approach we would recommend.

Perhaps surprisingly, readers’ clinical experience was not predictive of improved MDC. The reader with the lowest MDC% and the highest intra‐rater reliability for hemostatic efficacy category has been trained extensively in hemorrhage volume segmentation. Focused training on volume segmentation and retraining may be necessary to optimize reader's skills for hemorrhage volume measurements in clinical trials where hemostatic efficacy is a major endpoint. Such effort is critical to reducing the MDC% for each reader so that it falls below the excellent hemostatic efficacy threshold of 20%. Most readers were able to achieve an MDC% below such a threshold if total ICrH volume was used. Smaller ICrH volume was associated with higher measurement error when considering MDC%. Suggesting more care and attention to detail is needed when assessing hemostatic efficacy in patients with small ICrH volume.

Our results pose multiple similarities with lung nodule volumetric CT studies. First, smaller lung nodule measurements produced greater variability, than larger ones (Oxnard et al., 2011), similar to our findings. Furthermore, 20% seems to be the recommended limit for detectable growth in subcentimeter lung nodules (Gavrielides et al., 2013) which is similar to our results. Finally, ground glass opacities, which are often hazy and have unclear borders, pose a particularly challenging task to measure (Oda et al., 2010), which may parallel with the SAH compartment that is often hazy and subtle with unclear borders.

Limitations of our study include the wide range of intracranial bleeding evaluated. Several patients included in the ANNEXA‐4 trial had traumatic, complex multi‐compartmental bleeds, which were challenging to measure with a high degree of precision. In particular, the dataset used to assess aim two included several SDHs, which have been shown in this study to be quite difficult to measure precisely. The patient data used in aims one and two were from the same trial but given the random selection of each dataset, involved different subjects, which limits the applicability from one aim to the other.

## 5 CONCLUSION

Our results suggest that measuring total ICrH volume as a sum of all compartments is a more precise measure of hemostatic efficacy than by individual compartments. We also suggest that readers undergo training before measuring hemorrhage volumes for clinical trials to ensure that their MDC is lower than the suggested efficacy threshold, as intra‐rater reliability may improve with training. These findings can inform the design of studies assessing hematoma expansion as a predictor of outcome in patients with ICrH.

## AUTHOR CONTRIBUTIONS

**MacKenzie Horn**: Conceptualization; data curation; formal analysis; investigation; methodology; project administration; software; validation; writing—original draft. **Ankur Banerjee**: Conceptualization; data curation; formal analysis; investigation; methodology; writing—original draft. **Linda Kasickova**: Conceptualization; data curation; investigation; methodology; writing—review and editing. **Ondrej Volny**: Data curation; investigation; methodology; writing—review and editing. **Hyun Choi**: Data curation; investigation; methodology; writing—review and editing. **Federica Letteri**: Data curation; investigation; methodology; writing—review and editing. **Tomoyuki Ohara**: Data curation; investigation; methodology; writing—review and editing. **Koji Tanaka**: Conceptualization; data curation; formal analysis; investigation; methodology; writing—original draft. **Stuart Connolly**: Conceptualization; data curation; resources; supervision; writing—review and editing. **Per Ladenvall**: Conceptualization; data curation; resources; supervision; writing—review and editing. **Mark A. Crowther**: Conceptualization; data curation; resources; supervision; writing—review and editing. **Jan Beyer‐Westendorf**: Conceptualization; data curation; resources; supervision; writing—review and editing. **Ashkan Shoamanesh**: Conceptualization; data curation; resources; supervision; writing—review and editing. **Andrew M. Demchuk**: Conceptualization; data curation; formal analysis; investigation; methodology; project administration; resources; software; supervision; writing—original draft. **Abdulaziz Al Sultan**: Conceptualization; data curation; formal analysis; investigation; methodology; project administration; resources; software; supervision; writing—original draft.

## FUNDING INFORMATION

There was no funding obtained for this project.

## CONFLICT OF INTEREST STATEMENT

AB, MH, LK, OV, HC, FL, TO, KT, and AAS have no disclosures. SC has received grant support and served as a consultant for Portola, Bristol Myers Squibb, Bayer, and Daiichi Sankyo; has served as a consultant for Javelin; and holds a patent (US 2010/0255000) and pending patent (US 2017/0369862 A1). PL is an employee of AstraZeneca. MC received grants from Bayer AG; and personal fees from Servier Canada, Asahi Kasei, Precision Biologics, Hemostasis Reference Laboratories, Syneos Health, Pfizer Canada, CSL Behring, and Diagnostica Stago. JBW has received personal honoraria and travel support from Bayer, Daiichi Sankyo, Janssen, Sanofi, and Portola/Alexion; and institutional research support from Bayer, Daiichi Sankyo, Janssen, LEO, Pfizer, and Portola/Alexion. AS has received grants and advisory honoraria support from AstraZeneca. AMD has received honoraria from Hoffmann‐LaRoche for advisory board consultation; honoraria from Boehringer Ingelheim and Medtronic for CME lectures; is a patent/shareholder in Circle CVI; serves as a consultant, on the adjudication, and steering committees for ANNEXA‐4 for Astra Zeneca; and has received personal fees from Astra Zeneca.

### PEER REVIEW

The peer review history for this article is available at https://publons.com/publon/10.1002/brb3.3481.

## Data Availability

The data that support the findings of this study are available from the corresponding author upon reasonable request.
